# DenseIncepS115: a novel network-level fusion framework for Alzheimer's disease prediction using MRI images

**DOI:** 10.3389/fonc.2024.1501742

**Published:** 2024-12-03

**Authors:** Fatima Rauf, Muhammad Attique Khan, Ghassen Ben Brahim, Wardah Abrar, Areej Alasiry, Mehrez Marzougui, Seob Jeon, Yunyoung Nam

**Affiliations:** ^1^ Department of Computer Science, HITEC University, Taxila, Pakistan; ^2^ Department of Artificial Intelligence (AI), College of Computer Engineering and Science, Prince Mohammad Bin Fahd University, Al Khobar, Saudi Arabia; ^3^ College of Computer Science, King Khalid University, Abha, Saudi Arabia; ^4^ Department of Internal Medicine, Soonchunhyang University Cheonan Hospital, Cheonan, Republic of Korea; ^5^ ICT Convergence Research Centre, Soonchunhyang University, Asan, Republic of Korea

**Keywords:** neuroimaging, Alzheimer's disease, MRI, network-level fusion, multiscale inception module, dementia stages classification, optimization, shallow neural network

## Abstract

One of the most prevalent disorders relating to neurodegenerative conditions and dementia is Alzheimer's disease (AD). In the age group 65 and older, the prevalence of Alzheimer's disease is increasing. Before symptoms showed up, the disease had grown to a severe stage and resulted in an irreversible brain disorder that is not treatable with medication or other therapies. Therefore, early prediction is essential to slow down AD progression. Computer-aided diagnosis systems can be used as a second opinion by radiologists in their clinics to predict AD using MRI scans. In this work, we proposed a novel deep learning architecture named DenseIncepS115for for AD prediction from MRI scans. The proposed architecture is based on the Inception Module with Self-Attention (InceptionSA) and the Dense Module with Self-Attention (DenseSA). Both modules are fused at the network level using a depth concatenation layer. The proposed architecture hyperparameters are initialized using Bayesian Optimization, which impacts the better learning of the selected datasets. In the testing phase, features are extracted from the depth concatenation layer, which is further optimized using the Catch Fish Optimization (CFO) algorithm and passed to shallow wide neural network classifiers for the final prediction. In addition, the proposed DenseIncepS115 architecture is interpreted through Lime and Gradcam explainable techniques. Two publicly available datasets were employed in the experimental process: Alzheimer's ADNI and Alzheimer's classes MRI. On both datasets, the proposed architecture obtained an accuracy level of 99.5% and 98.5%, respectively. Detailed ablation studies and comparisons with state-of-the-art techniques show that the proposed architecture outperforms.

## 1 Introduction

Brain-related disorders are among the most challenging conditions due to their high cost, their sensitivity, and the difficulty in treating them. The most prevalent brain disease affecting people is Alzheimer's disease, which causes various degrees of memory loss and knowledge loss (1). According to the most recent World Alzheimer Report (2), there are 55 million clinically diagnosed AD patients worldwide, and by 2050, that number is expected to reach 139 million (3). People over the age of 65 are the most likely to suffer from this irreversible disorder (4). Alzheimer's disease is more prevalent in individuals with diabetes, cardiovascular issues, and hypertension. This neurological condition begins slowly and gets worse every day. The early signs and symptoms of Alzheimer's include memory loss, difficulties completing daily tasks, confusion about where you are, visual or spatial difficulties, language difficulties, poor decisions, withdrawal from work, mood swings like depression, as well as behavioral and personality changes (5). As a result of the most recent advances in multimodal neuroimaging data, early disease detection has been enabled in neuroscience (6, 7). The pattern of brain shrinkage and image intensities, however, are so similar that it has been difficult to distinguish between healthy and Alzheimer's brains (8). The body gradually loses its ability to function, which may finally result in death, even though the exact cause of this disorder is unknown (9). AD does not have a cure at present, but if detected early, its progression may be slowed.

In order to diagnose brain disorders, neuroimaging techniques such as magnetic resonance imaging (MRI) (10) or computed tomography (CT) (11) with positron emission tomography (PET) (11) use images to provide three-dimensional (3D) images of the brain (12). It was estimated that AD patients would live only 3.1 years, especially if they were diagnosed at an early stage. Mild cognitive impairment (MCI) is a state of amnesia that may be an early indicator of Alzheimer's disease (13), but it is constantly getting worse. Non-sympathetic (generalized psychosis) Alzheimer's disease progresses in three stages: mild (stage 1), severe (stage 2), and moderate dementia (stage 3) (14). It is essential for AD diagnoses to be automated since clinical treatments are highly expensive (15). Machine learning (ML) paradigms have been expanded with new methods for learning in healthcare, especially for medical imaging (16). The traditional ML techniques focused on handcrafted features such as shape, color, and geometric; however, the complex imaging datasets required more patterns for an accurate prediction. Issues in traditional ML techniques include extra middle steps for the extraction of individual patterns, large number of extracted patterns (extracted features), and irrelevant information extraction.

**Figure 1:**
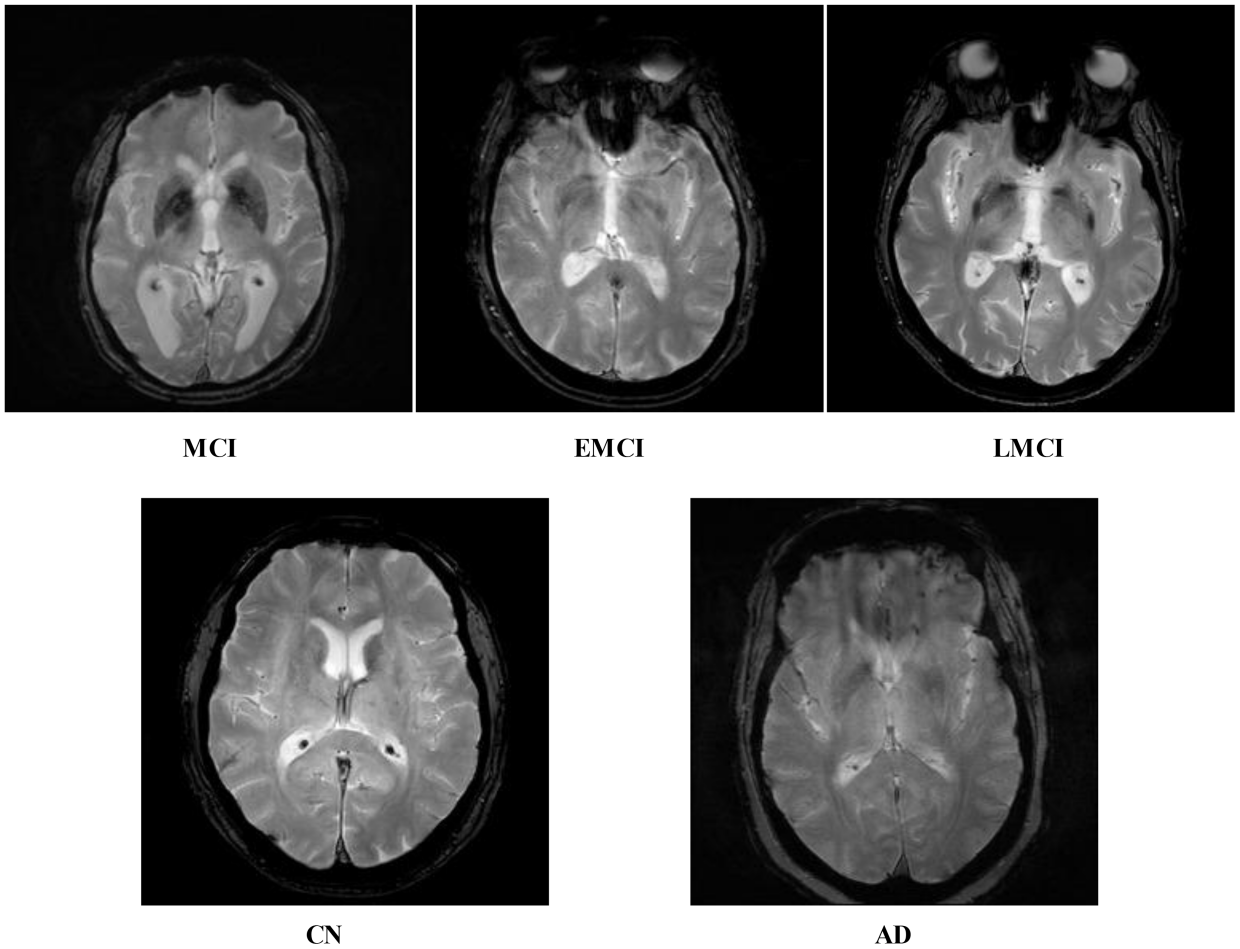
Five different categories of ADNI dataset: Alzheimer's disease (AD), cognitively normal (CN), early mild cognitive impairment (EMCI), late mild cognitive impairment (LMCI), and subjective memory complaint (MCI).

Recent advancements in the area of deep learning (DL) techniques are becoming more and more popular in several applications, especially in medical imaging (17, 18). Convolutional neural networks (CNN), a specialized DL method, significantly outperform state-of-the-art ML techniques in various applications (19). These studies highlight CNN's superior ability to automatically extract and learn complex features from data, leading to improved accuracy and performance in tasks such as image recognition, classification, and other predictive modeling scenarios (20, 21). The DL algorithms work based on the hidden layers such as convolutional layer, pooling layer, and fully connected layers (22). There are several pre-trained DL architectures available for classification purposes such as GoogleNet (23), Alexnet (24), VGG (25), ResNet, and Inception-ResNet (25). Each model has a different way of learning mechanism. These models performed well for several classification tasks; however, in medical imaging, it degrades the performance when there is an issue of data imbalance and complex patterns of the disease. In addition, these models degraded the performance due to similar patterns of different classes, as shown in **Figure 1**. Therefore, for the customized models, it is always required that these are designed from scratch for a specific problem (26). The customized CNN models are usually designed based on the structure of the hidden layers and the number of learnable (27). This work proposed a novel network-level fused deep architecture based on dense and inception modules with self-attention mechanism for the classification of brain MRIs to predict and diagnose Alzheimer's disease. In the proposed network, deep features are extracted from brain MRI images using DenseNet and multi-scale Inception modules. These architectures are further integrated at the network level and can accurately predict patients with AD such as EMCI, MCI, and LMCI and those who are cognitively normal.

Following is a summary of the major contributions of this work:

- Develop a novel network-level fused convolutional neural network architecture called Dense with Multiscale Inception Self Module (DenseIncepS115) for the classification of Alzheimer's disease.
- The inception module with multiscale heads is designed to improve model accuracy and generalization. The concept of inverted bottlenecks is also considered to optimize network learning on the selected datasets.
- To increase model robustness, a data augmentation technique is also designed based on color enhancement.
- Extracting deep features with the use of multi-head self-attention mechanisms that are later optimized using Catch Fish Optimization Algorithm (CFOA).
- Demonstrating the efficiency of the proposed architecture, several ablation studies have been performed such as computed results using several pre-trained networks, selection of learning rate through manual approach, and confidence interval-based analysis.

## 2 Literature review

Alzheimer's disease has been attributed to genetics, but its underlying causes remain unknown. There are numerous issues related to social cognitive abilities that are affected by Alzheimer's, which also cause a number of neurological issues related to memory (28). Sarraf et al. (29) discussed about the very first use of MRI data in deep learning applications for Alzheimer's disease prediction and medical image analysis. The suggested pipelines produced average accuracy rates of 94.32% for fMRI and 97.88% for MRI, whereas the high accuracy rate of 98.84% for MRI was attained by subject-level classification. Hamdi et al. (12) addressed the problem of differentiating Alzheimer's disease patients from normal controls by developing a novel and improved CAD system based on a convolutional neural network (CNN). Using the 18FDG-PET images of 855 patients—220 Alzheimer's disease patients and 635 normal controls—from the ADNI database, the presented method was assessed. The findings demonstrated that the CAD system produced 96% accuracy, 96% sensitivity, and 94% specificity, respectively. Zhang et al. (30) developed an attention-based CNN that was trained using multilevel data from brain MRI to classify Alzheimer's disease. Using the ADNI dataset, this method identified Alzheimer's disease patients with 97.35% accuracy, MCI converters with 87.82% accuracy, and non-converters with 78.79% accuracy, respectively.

Divya et al. (31) utilized MRI features from the ADNI dataset to classify normal control (NC), mild cognitive impairment (MCI), and Alzheimer's disease (AD) using feature selection techniques and supervised learning algorithms. The best results were obtained with a support vector machine (SVM) with a radial basis function kernel, achieving 96.82%, 89.39%, and 90.40% accuracy for binary classifications of NC/AD, NC/MCI, and MCI/AD, respectively. The LeNet model was modified in this study (32) by concatenating Min-Pooling layers with Max-Pooling layers so that low-intensity pixels are preserved. This new model performed best when compared with 20 different DNN models, with an average accuracy of 96.64% for Alzheimer's disease classification compared to 80% for the original LeNet.

Shamrat et al. (33) developed a fine-tuned CNN architecture for Alzheimer's disease prediction into five stages. They modify the model based on the layers and hyperparameters. After that, they used 60,000 MRI scans from the ADNI database and obtained highest accuracy of 96.31%. Tanveer et al. (34) presented a computationally efficient ensemble of neural networks trained with transfer learning. The classification accuracy for mild cognitive impairment (MCI) vs. Alzheimer's disease (AD) was 98.71% and 83.11%, respectively, on two independent datasets split by cognitively normal (NC) vs. AD. Hajamohideen et al. (35) developed an architecture for a Siamese Convolutional Neural Network (SCNN) which embeds input images as k-dimensional embedding's with a triplet loss function. This embedding space was used to classify Alzheimer's disease using both pre-trained and non-trained CNNs. Model effectiveness was evaluated using ADNI and OASIS datasets, which yielded accuracy rates of 91.83% and 93.85%, respectively.

Alp et al. (36) investigated the use of Vision Transformer (ViT) for processing MRIs in the diagnosis of Alzheimer's disease. ViT used as a time series transformer to classify the MRI features once they had been extracted and modeled. On ADNI T1-weighted MRIs, the model was evaluated for binary and multiclass classification. In comparison to deep learning models such as CNN with BiL-STM and ViT with Bi-LSTM, the model demonstrated a high level of accuracy, scoring above 95% for binary and 96% for multiclass classification. Shaffi et al. (37) offered an ensemble classifier based on machine learning for the prediction of AD from MRI scans. It demonstrated an amazing accuracy of 96.52%, which is 3-5% better than the best individual classifier. They used the Alzheimer's Disease Neuroimaging Initiative and Open Access Series of Imaging Studies datasets to assess well-known machine learning classifiers and obtained improved accuracy above 94%. The study (16) suggested a bilateral filtering and histogram equalization image enhancement strategy to enhance the quality of the dataset. Then, to classify dementia into three groups, a custom CNN architecture has been designed. Using the designed custom architecture, the presented architecture obtained an accuracy of 93.45% and 95.62% for multiclass and binary class, respectively.

In summary, the above mentioned studies focused on the pre-trained models and supervised learning algorithms. In addition, they also focused on the traditional augmentation techniques such as flip and rotate methods. These methods not focused on the networks fusion and features optimization. Also, they did not focus on the shallow neural network classifiers for the prediction of AD from the MRI scans. In this work, we proposed a novel network-level fused CNN architecture for the prediction of AD from the MRI scans.

## 3 Proposed methodology

**Figure 2:**
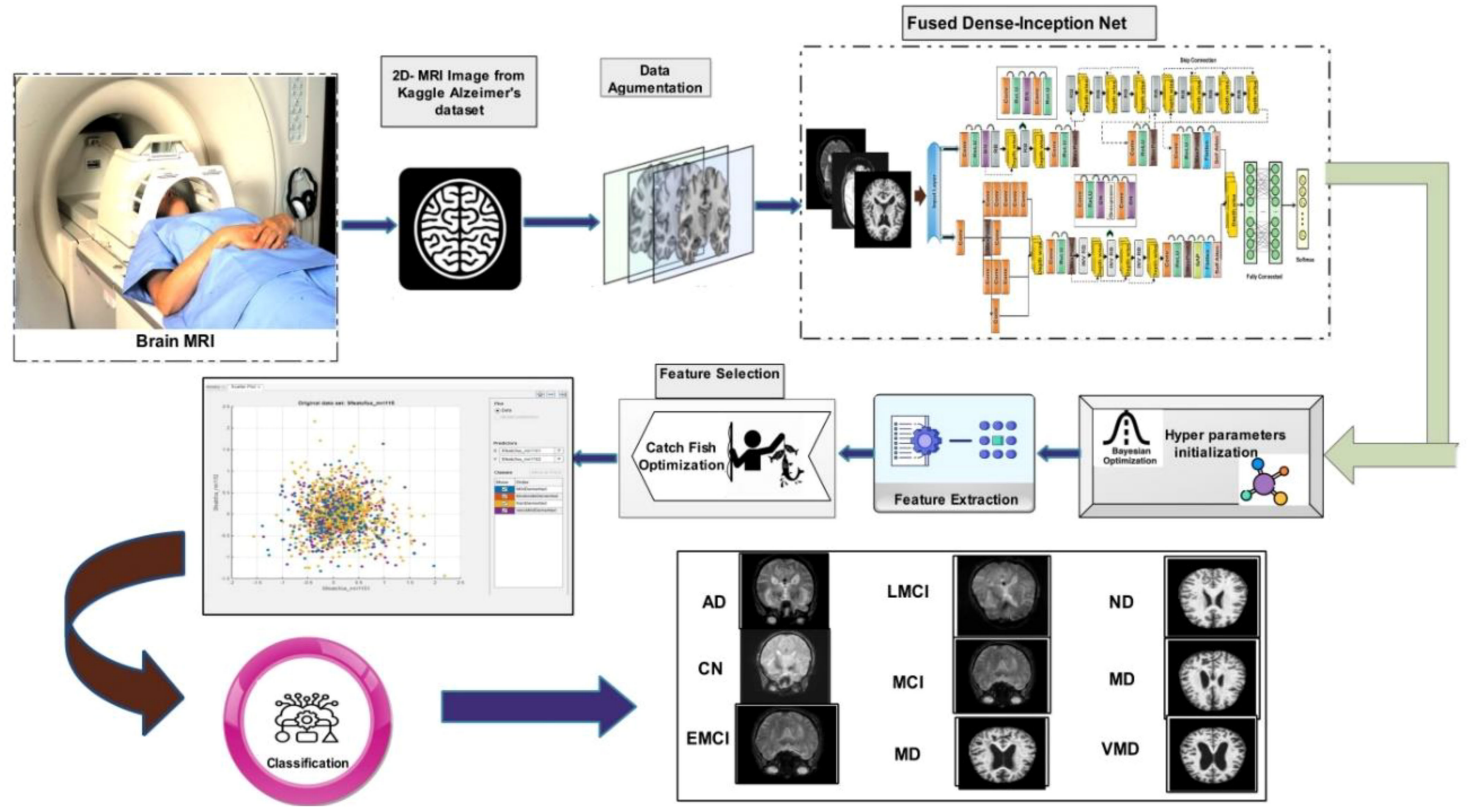
Proposed automated framework for classifying Alzheimer's disease using network-level fusion.

In this work, we proposed novel DenseIncepS115 architecture for the prediction of Alzheimer's disease from MRI scans. The proposed architecture comprises two novel modules—inception module with multiscale self-heads and dense module with self-attention. **Figure 2** illustrates the proposed architecture of Alzheimer's disease prediction. In the proposed architecture, a dataset augmentation step has been performed at the first step to increase the training diversity. In the second step, both modules are fused at the network level using depth-concatenation layer. The model is trained on the selected dataset, whereas Bayesian optimization (BO) has been opted for the hyperparameters' initialization. In the testing phase, features are extracted from the testing data and optimized using an improved Catch Fish Optimization Algorithm technique. The best features are selected and passed to the shallow wide neural network (SWNN) classifier for final classification. Furthermore, the fused model is interpreted using GRAD-CAM and LIME explainable techniques.

### 3.1 Dataset collection and augmentation

**Figure 3:**
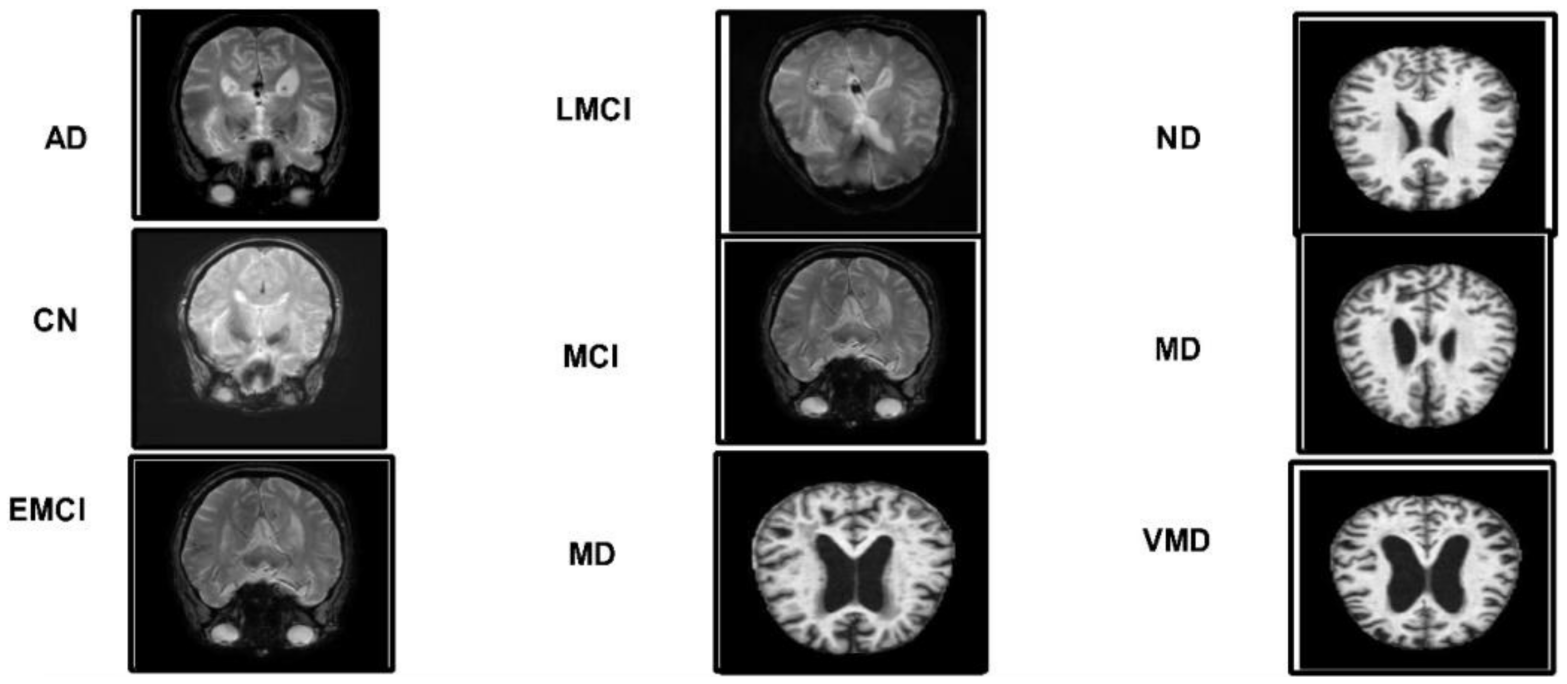
A few sample images of the selected Alzheimer's datasets.

ADNI dataset: Data from the Alzheimer's disease neuroimaging initiative (ADNI) database were used in this investigation. In 2003, the ADNI was developed as a public–private partnership under the direction of principal investigator Michael W. Weiner, MD. The purpose of this project was to explore the potential utility of positron emission tomography (PET), magnetic resonance imaging (MRI), and other biological markers in monitoring the development of early diagnosis of Alzheimer's disease and mild cognitive impairment (38). The dataset used in this work, which was collected from Kaggle, contains five categories: AD, CN, EMCI, LMCI and MCI genetic information, and the results from cognitive tests are included in the ADNI collection (39). **Figure 3** illustrates the sample images of this dataset. The dataset contains both and female patients, wherein 100 out of 416 patients aged 60+ have been diagnosed with AD, ranging from very mild to moderate. A brief description of the dataset is given in **Table 1**.

**Table 1:** Description of selected Alzheimer MRI dataset for this paper.

S. no.	Classes	Original images	Augmentation (training/testing)
Alzheimer ADNI 5-classes MRI dataset			
1	AD	728	4,004/364
2	CN	696	4,004/348
3	EMCI	720	4,004/360
4	LMCI	704	4,004/352
5	MCI	700	4,004/350
Alzheimer MRI 4-classes dataset			
1	Mildly demented (MD)	1,075	2,560/537
2	Moderately demented (MoD)	756	2,560/120
3	Non-demented (ND)	3,200	2,560/1,280
4	Very mildly demented (VMD)	2,240	2,560/896

Alzheimer MRI dataset: The AD preprocessed magnetic resonance imaging (MRI) images (ADMPIs) make up the dataset. The dataset contains four classes such as mildly demented, moderately demented, non-demented, and very mildly demented. The image details in each class are given in **Table 1**. In this work, 1,564 ADMPIs were used from the Kaggle source (40). A few sample images are shown in **Figure 3**.

During dataset collection, it was observed that the samples in both datasets have a class imbalance problem and are insufficient to train a deep learning model efficiently. This can lead to biases and overfitting during the model's training. To address these challenges, we performed an augmentation process to normalize the samples in each class and increase the diversity in the selected datasets. In the augmentation step, we performed three operations, such as flip left, flip right, and rotations. The overall description of the datasets is presented in **Table 1**.

### 3.2 Proposed DenseIncepS115 architecture

Deep learning is an important research area in the area of computer vision for classification and detection tasks. Medical imaging is an important research area, and many deep learning techniques are introduced for the classification and detection of medical diagnosis such as breast cancer, skin cancer, brain tumor, Alzheimer's disease (AD), and a few more. AD draws much attention from researchers working in the area of neuro-related diseases. Through DL, a better precision rate can be achieved for diagnosis and classification. In this paper, we proposed a novel DenseIncepS115 Architecture with explainable AI (XAI) for the classification and interpretation of AD from MRI scans. The dense and inception modules were chosen because the densely connected layers allowed the network to capture fine-grained information, such as subtle tissue degradations and gray matter changes, which indicate early Alzheimer's disease. In addition, inception modules have the capability to capture inclusive and specific regions, such as overall brain shrinkage and cortical folding–shifting in Alzheimer's disease. In this study, the capabilities of both modules are integrated using a network-level fusion method in the classification of Alzheimer's disease through the different phases.

**Figure 4:**
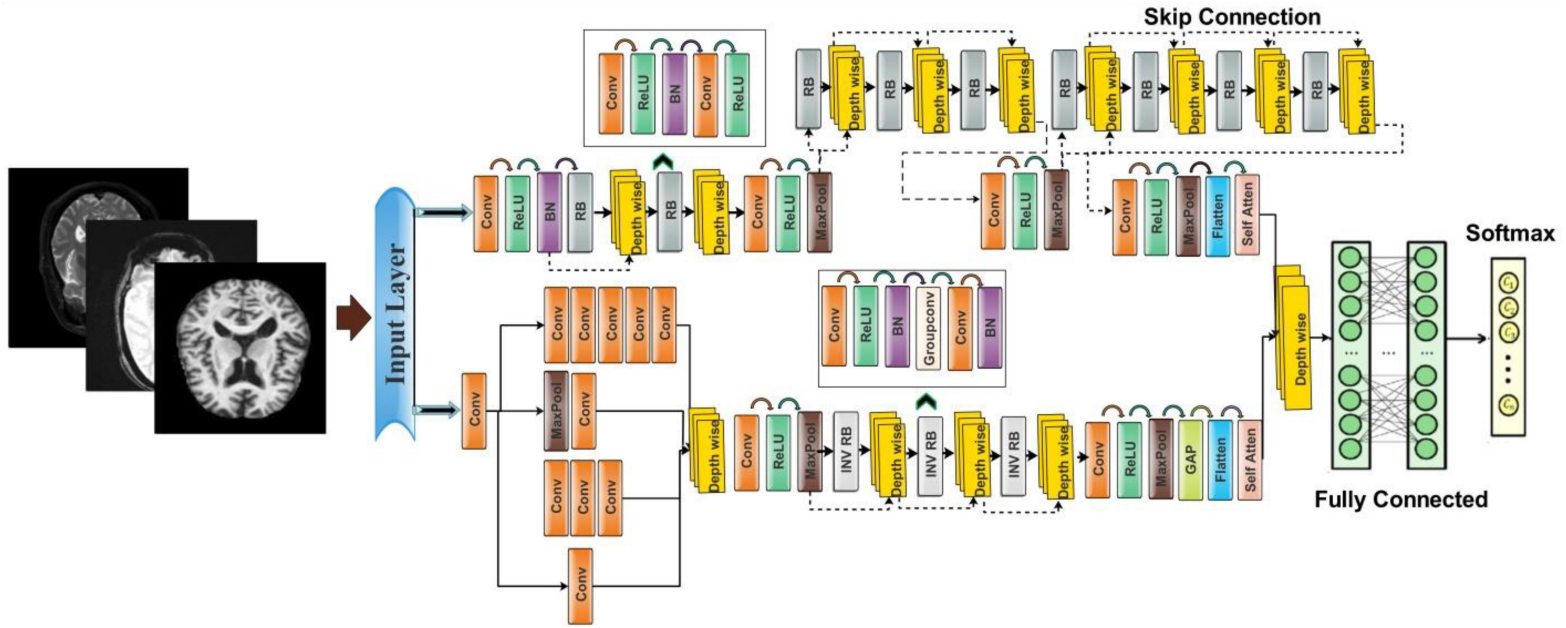
A visual architecture of the proposed DenseIncep-115 CNN.

The proposed architecture consists of two modules such as an inception module with multiscale self-heads and a dense module with self-attention. The total number of parameters of the proposed architecture is 6.9 million, whereas the total numbers of layers is 115. **Figure 4** shows the architecture of the proposed DenseIncepS115 model. The input size of the proposed architecture is 227 × 227 × 3.

Dense module with self-attention: The self-module consists of 71 layers, including nine residual blocks and 22 convolution layers. At the start of the architecture, a convolutional layer has been added with a 3 × 3 filter size, stride value of 2, and depth of 32. Then, ReLU activation has been added as a nonlinearity function that attached with the batch normalization layer. After this, a first residual block has been added. Each block contains five layers such as convolutional layer, BN (batch normalization), ReLU, convolutional layer, and ReLU layers. In the first residual block, the first convolutional layer has a depth size of 64 with a stride value of 1 and 1×1 filter size. A ReLU activation layer is added with the batch normalization layer. The first residual block concludes by adding the last convolutional layer with depth of 64, stride value of 1, and filter size of 1 × 1. Then, these residual blocks are connected to the other layers by adding the first depth concatenation layer that concatenate inputs along the channel dimension which involves taking inputs with the same height and width.

The second residual block is added in same layer pattern in which the convolutional layers have a depth size of 96 and 64 with a stride value of 1 and filter size 1 × 1. Moreover, a depth concatenation layer has been added to connect it with the other layers, and there is a skip connection between both depth concatenation layers. A convolutional layer with ReLU has been added after this, with filter size of 2 × 2 and stride value of 2. The depth size is increased in this block to 128 from what was previously 96. A max-pool layer is added after this to get the most activated neurons with a stride value of 2 and filter size of 3 × 3. Three more residual blocks are added after the max-pooling layer. All of these blocks are connected with skip connection through the depth concatenation layer. The number of layers in each block is five, which is similar to the first residual and second residual block. The convolutional layers in the first block have a depth size of 128 and filter size of 1 × 1. In the second block, the convolutional layers have a depth size of 128 and 64, whereas the stride is 1 and the filter size is 1 × 1. A third block follows the first block, of which both convolutional layers have depth of 128, filter size of 1 × 1, and stride of 1. After that, a convolutional layer with ReLU is added, which has a depth of 256, a filter size of 2 × 2, and a stride value of 2. Another max-pool layer has been attached with a filter size of 3 × 3 and a stride value of 2.

**Figure 5:**
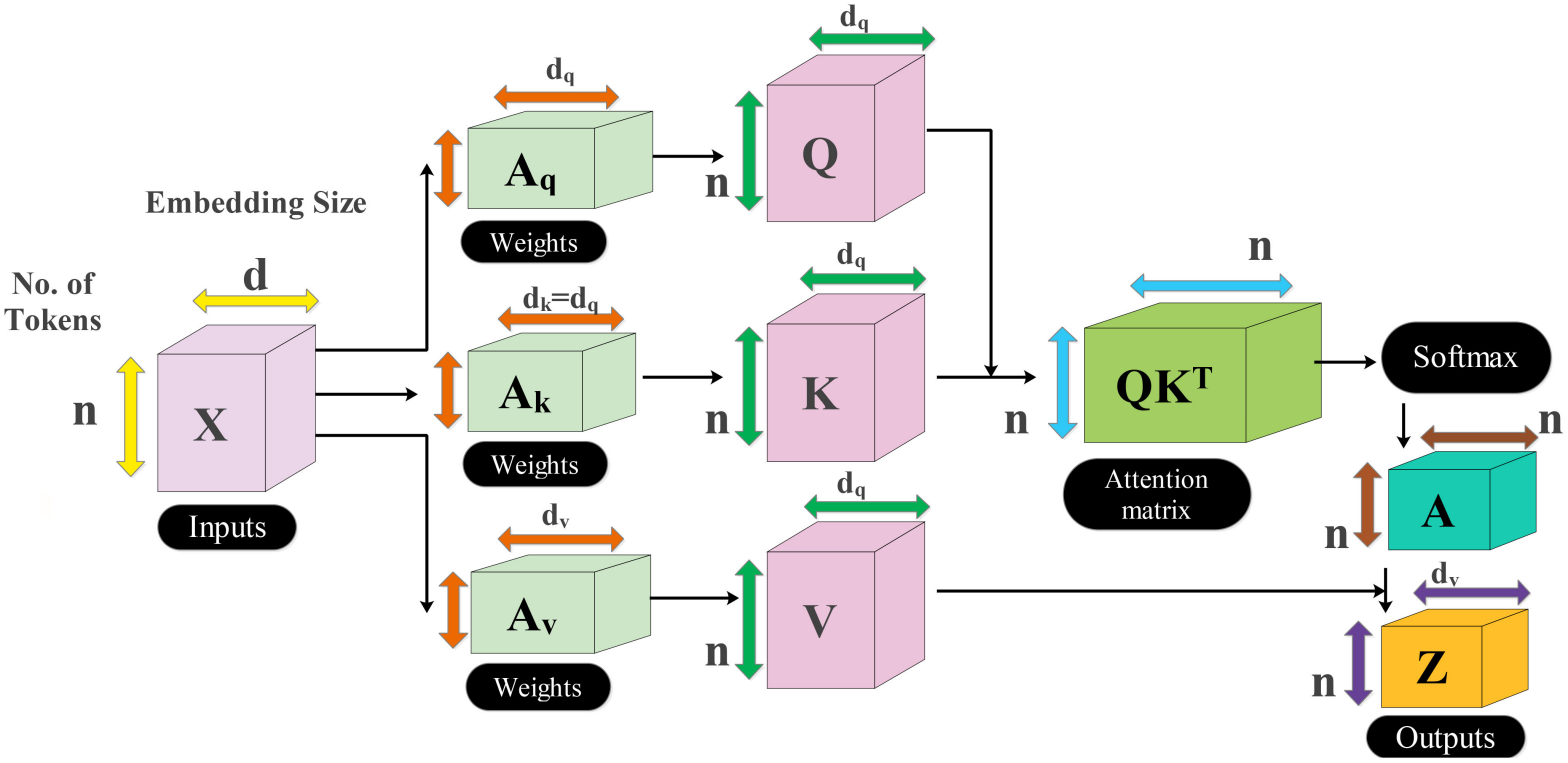
Visual illustration of self-attention for in-depth feature extraction from MRI scans.

There are four more residual blocks attached to the network after the second max-pool layer that are connected via depth concatenation. The max-pooling layer and the depth concatenation layer, as well as the remaining four depth concatenation layers, are interconnected by skip connections. These blocks follow the same number of layers (five) as the previous blocks in the same pattern but with different parameters. In the first block, both convolutional layers have a depth of 256 with different filter sizes of 1 × 1 and 3 × 3 and a stride value of 1 × 1. With the second residual block, both convolutional layers have a depth size of 128 and constant filter sizes of 1 × 1 and stride of 1. The third block follows the first block in which both convolutional layers have depth of 256 and a stride value of 1 with the same filter size of 1 × 1. At the end, a convolutional layer has been added that has a filter size of 2 × 2, a stride value of 2, and a depth size of 256. Another max-pool layer is added with a filter size of 3 × 3 and a stride of 2. After that, a self-attention module is added for the in-depth information extraction of each image. The self-attention module is visually shown in **Figure 5**. The self-attention module is mathematically formulated as follows:

As the inputs of attention module are queries, keys, and values which are defined with $A_{q}$ , $A_{k}$ , and $A_{v}$ , respectively, these notations created a linear transformation as follows:


(1) $$A_{q}=M\;X_{A_{q}}$$

(2) $$A_{k}=M\;X_{A_{k}}$$

(3) $$A_{v}=M\;X_{A_{v}}$$

where M denotes the input feature matrix of the convolutional layer and $M\in {\mathbb{R}}^{m\times n}$ . After that, the attention score is computed among $A_{q}$ and $A_{k}$ using a dot product, which is later scaled down by the factor of $\sqrt{d_{k}}$ . This scaling is performed to prevent the dot product from growing too large during the training process. The attention score can be formulated by using the following equation:


(4) $$AtS=\frac{qk^{T}}{\sqrt{d_{k}}}$$

The computed score AtS is passed to softmax function to obtain the final attention weights as follows:


(5) $$AtW=Sfmx\left(AtS\right)$$

On each row of the attention score, the softmax function has been applied, which sums up to 1 for the final score. Hence, the weighted sum is applied to create the attention weights as final features.


(6) $$Attn\;\left(A_{q},A_{k},\;A_{v}\right)=\left(AtW\right)v$$

Inception module with multiscale self-heads: The proposed inception module with multiscale self-heads consists of 44 layers and follows the inverted bottleneck pattern in residual blocks. The first convolutional layer has a depth size of 16 and a filter size of 1 × 1 with a stride value of 2. Then, the first inception module has been added, in which the first convolutional layer is added with a depth value of 16 and a filter size of 1 × 1. The second convolutional layer also has a depth size of 16 and a filter size of 1 × 1 with a single stride. For the third convolutional layer, a depth size of 32 is opted, with a filter size of 1 × 1 and a stride value of 1. In the next or third convolutional layer, a max-pool layer has been added with a filter size of 3 × 3 and a stride value of 1. The first convolutional layer is followed by a second convolution layer with a depth size of 16 and a filter size of 1 × 1. The output is divided in two convolutional layers of filter size 1 × 3 and 3 × 1, respectively. Moreover, 1 × 1 and 3 × 1 filters are added before the max-pooling layer of filter size 1 × 1. When convolutions do not significantly change the dimensions of the input, neural networks performed better work and reduced the dimensions. An excess of small dimensional reductions may result in information loss; therefore, convolutions and smart factorization techniques can be made more computationally efficient. This module is connected by adding one depth concatenation layer. After that, a convolutional layer with ReLU is added, which has a filter size of 3 × 3 with a stride value of 2 and depth of 64. A max-pool layer that has a stride value of 1 and filter size of 3 × 3 was attached.

Three inverted residual blocks are added after the inception module, where each block consists of six layers such as convolutional, ReLU, batch normalization, grouped convolutional, convolutional, and batch normalization layer. In the first block, the convolutional layer has a depth size of 128, with a filter size of 1 × 1 and a stride value of 1. Then, a batch normalization layer is added with ReLU activation. After that, the grouped convolutional layer is added with a 3 × 3 filter size. The first residual block concludes by adding the last convolutional layer with a depth of 256, a stride value of 1, and a filter size of 1 × 1. These residual blocks are connected to the other layers by adding the first depth concatenation layer. The second block is identical to the previous block with the same parameters. In the third block, convolutional layer with a filter size of 1 × 1 was employed, with a stride value of 1 and depth size of 256 and 312, respectively. After that, a convolutional layer with ReLU is added, which has a filter size of 3 × 3 and a stride value of 2 with a depth of 256. A max-pooling layer has been added with 3 × 3 filter size that followed a global average pool layer. At the end, we added a multi-headed self-attention module, as illustrated in **Figure 5**. The flattened layer is always added before the self-attention module.

The outputs of both modules are fused at the network level using a depth concatenation layer, as shown in **Figure 4**. Through the depth concatenation layer, information of different layers can be stacked into a single layer based on the depth dimension. It enriches the information in the form of feature vector and, as an output, accurate AD prediction.

Consider that we have two feature vectors denoted by $f_{1}$ and $f_{2}$ of dimensions N×256 and N×256 , respectively. The depth concatenation (DC) is formulated through the following equation:


(7) $$DC=DepthCAT\left(f_{1},\;f_{2}\right),\;\text{where,}\;DC=h\times w\times \left(d_{1}+d_{2}\right)$$

Here $d_{1}$ and $d_{2}$ denote the depth of both feature vectors that is 256. Hence, the final feature vector dimension after the DC layer is 512, which passed to the fully connected layer and softmax for the final classification. The softmax is employed as a classification layer in the proposed architecture. Mathematically, the combined loss function of softmax and cross entropy is defined as follows:


(8) $$loss=-\log(\frac{e^{t_{y}}}{\sum_{j=1}^{Cl}e^{t_{j}}})$$

(9) $$=-t_{y}+\log(\sum_{j=1}^{Cl}e^{t_{j}})$$

where $t_{y}$ denotes the raw score for the true class. The detailed architecture is described under **Algorithm 1**.

Algorithm 1 **Pseudocode for DenseIncepS115 architecture.**

**1: Input:** 

$D_{A}$

: Alzheimer's Training Set; 

$A_{L}$

: Actual Labels
**2: Output:** Trained DenseIncepS115 Model
**3:** dataset split: 

$D_{train},D_{validation}\leftarrow split\left(D_{A},0.60,0.40\right)$


4: 

$Aug_{train},Aug_{validation}\leftarrow Transform\left(\;D_{train},D_{validation},\;Resize,\;transformations\right)$



**5: DenseIncepS115 architecture:
**
 **1: Input image size:** 227×227×3 **2: Dense Module with Self-Attention:**
  **1: Convolutional layer:** Filters: 32, Size: 3×3, 
  Stride: 2  **2: ReLU+ BN**
  **3: Residual Block (1^st^ Block)**
   Convolution Layer: Filters: 64, Size: 1×1, 
   Stride: 1   Batch Normalization + ReLU   Convolution Layer: Filters: 64, Size: 1×1, 
   Stride: 1   Depth Concatenation Layer + Skip Connection  **4: Add Residual Block (2^nd^ Block)**
   Same pattern as above with filters adjusted to 96 
   and 64   Convolution Layer: Filters: 128, Size: 2×2, 
   Stride: 2   ReLU + Max Pooling (Filter: 3×3, Stride: 2)  **5: Add Three more Residual Blocks**
   Each with adjusted filter sizes (128, 64, 256)   Depth Concatenation Layer  **6: Max Pooling Layer:** Size: 3×3, Stride: 2  **7: Add Four more Residual Blocks**
   Adjust filter sizes with varying patterns (256, 
   128)   Skip Connections and Depth Concatenation Layer  **8: Flatten**
  **9: Self-Attention** 

$S_{D\;}$

 **(Dense Module)**
 **3: Inception Module with Multiscale Self-Attention:**
  **1: Convolution Layer:** Filters: 16, Size: 1×1, 
  Stride: 2  **2: Inception Block**
   Multiple convolutional paths (1×1, 3×1, 1×3 
   filters)   Depth Concatenation of paths  **3: Max Pooling + Convolution Layer:** Filters: 64, 
  Size: 3×3, Stride: 2  **4: Add three Inverted Residual Blocks**
   Each with convolution, grouped convolution 
   adjusted (128,256, 312)   ReLU Activation   Depth Concatenation Layer  **5: Global Average Pool Layer**
  **6: Flatten**
  **7: Self-Attention** 

$S_{I\;}$

 **((Inception module)**
 **4: Depth-wise Concatenation ((Network level Fusion)**
  **1:** 

$\varphi_{NL}\leftarrow Concatenation\left(S_{D},S_{I}\right)$



**5: Fully Connected (*number of Classes*)**
 **6: Softmax**
 **7: Classification**

**6:** 

$,\;\tau_{hyperparameters}\leftarrow Hyperparameters\left(\alpha ,\omega ,Aug_{validation},\epsilon \right)$


  

∴ α learning Rate;ω:mini batch size; ϵ validation frequency



**7: M**


$\leftarrow DenseIncepS115\left(Aug_{train},\;A_{L},\;\tau_{hyperparameters}\right)$

Training the proposed model: In training the proposed architecture, we used 70% of each dataset to train the model and the remaining 30% for the testing phase. In this process, several hyperparameters are required to initialize, such as initial learning rate, momentum, and batch size. These hyperparameters are usually initialized based on a random process or hit and trial; however, we employed Bayesian optimization (BO) (40) that returned the best-fit values after 100 iterations. There are a few other hyperparameters of this network, such as stride and filter size, that are selected based on literature knowledge. After the initialization process, the network has been trained with five folds and 100 epochs for each dataset.

### 3.3 Testing the proposed framework

**Figure 6:**
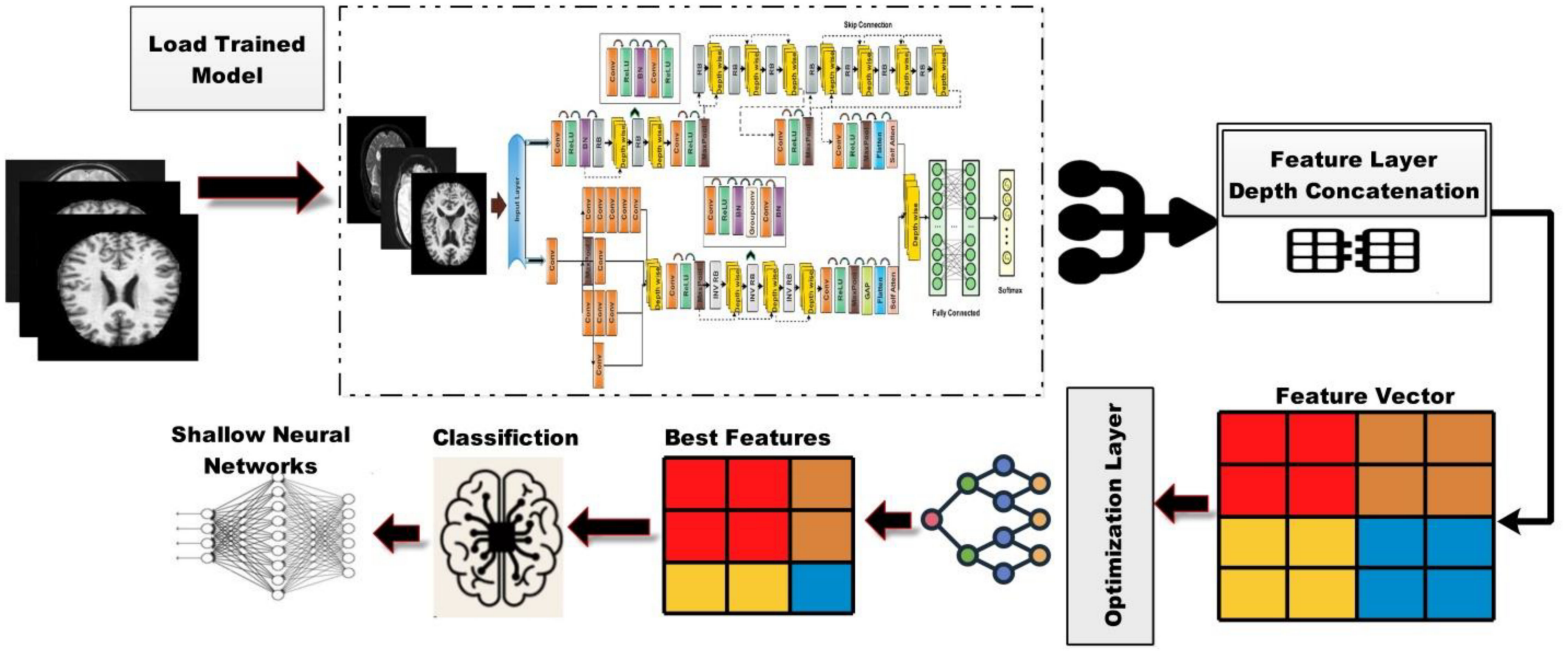
Visual illustration of the proposed architecture testing phase.

The proposed trained model DenseIncepS115 is tested on the testing image set and explained in this section. Deep learning features are extracted from the depth concatenation layer, and the performance was analyzed. In the analysis process, some redundant features are found; therefore, we employed a feature selection algorithm—Catch Fish Optimization (CFO). The CFO algorithm is applied in order to select the best features and pass them to the shallow wide neural network and traditional neural network classifiers. The shallow neural network has an advantage as it requires less computational power and is easier to train compared to deep neural networks. This simplicity often results in faster processing times, which is crucial for timely diagnoses. The visual illustration of the testing phase is shown in **Figure 6**.

### 3.4 Catch Fish Optimization Algorithm

The extracted features from Alzheimer's disease are often complex and multidimensional. The motivation behind choosing the CFO algorithm is due to its adaptive search; it allows broad exploration across all feature dimensions. Once a promising region is found, it can refine (exploit) that region to select the most appropriate features. This makes it effective to avoid local optima and ensure significant patterns, which other methods may have missed, and the complexity of Alzheimer's disease might vary considerably, enabling the algorithm to react dynamically to various features without requiring user intervention. This facilitates partial expansion throughout the feature selection process, ensuring that the optimal features are selected. After feature extraction, it is observed that the extracted information has some redundant features, which leads to the ineffective use of computational resources, and weak features do not contribute expressively to the model's decision. Therefore, we employed the CFO algorithm for optimal feature selection. The Catch Fish Optimization (CFO) algorithm introduced in (41) is based on the easy and traditional fishing technique called "fishing in water bodies," which is frequently used in rural regions. In the past, fishermen used the phrase "trouble the water to catch the fish" to describe a method of disrupting the water in a pond to confuse the fish into thinking that they were in clear water and to capture them easily. The basic tenet of CFOA is teamwork among members to maximize fish catches. It is possible for individuals to use tools and share stories about their individual experiences when they fish in diverse bodies of water. The optimization process is based on the following steps:

Step 1: The first step is to initialize the population. Every fisherman in CFOA serves as a search agent. Mathematically, it is defined as follows:


(10) $$Fisher=[\begin{array}{ccc} Fisher_{1,1} & \cdots Fisher_{1,2} & \cdots \;\;Fisher_{1,d}\\ Fisher_{\begin{array}{c} 2,1\\ \vdots \end{array}} & \cdots \;Fisher_{\begin{array}{c} 2,2\\ \ddots \end{array}} & \vdots \;Fishe_{\begin{array}{c} 2,d\\ \vdots \\ \vdots \end{array}}\\ Fisher_{M,1} & Fisher_{M,2} & \;Fisher_{M,d} \end{array}]\;M\times w$$

Fisher is a complete matrix of location data for M search agents in a space of dimension w . The locations can be updated based on the following formula:


(11) $$Fisher_{j,k}=\left(ub_{k}-lb_{k}\right)*n+lb_{k}$$

The location of the jth fisher in the kth dimension is denoted by $Fisher_{j,k}$ , the upper and lower limits of the kth dimension are denoted by $ub_{k}$ and $lb_{k}$ , and n is a random number between 0 and 1.

Step 2: In the second step, the fitness value and update fitness and optimal position are computed. The fitness values of each fisherman are determined via the fitness value evaluation function fobj using their current position information. Mathematically, fitness can be determined as follows:


(12) $$fit=fobj\;\left(Fisher\right)=\left[\begin{array}{c} fit_{1}\\ fit_{2}\\ \vdots \\ fit_{N} \end{array}\right]$$

The fitness values of the first and second fishermen are represented by $fit_{1}$ and $fit_{2}$ , respectively, in the fitness matrix. The ratio of exploration to exploitation is evenly distributed among the iterations using 0.5. In this procedure, global search was performed during the first phase (when EFs/MaxEFs<0.5 ) and exploited during the second phase (when EFs/MaxEFs >= 0.5 ).

Step 3: In this step, we determine whether exploration or exploitation is the present stage. This phase is based on two sub-stages.

a) Phase of exploration (EFs/MaxEFs<0.5): Update the position and randomly reshuffle each fisherman's position using the formulas (14)–(16) or (17) and (18). Fish population and capture rate drop with continuous fish capturing. Fishermen shift from solo exploration to group encirclement using individual skills in support. This transition can be modeled using the capture rate parameter, represented by â.


(13) $$\text{â}={\left(1-\frac{3\;\times EF_{s}}{2\;\times \;MaxEF_{s}}\right)}^{\frac{3\times EF_{s}}{2\;\times \;MaxEF_{s}}}$$

Fishermen have the option of either a group catch or an independent search. Individual search is preferred when the rate of capture â increases. They move to group capture as â decreases. Random integers (0, 1) are used to simulate this: when  ƿ<â , independent search is selected, and when ƿ≥â , group capture is selected. Here ƿ is a random value between 0 and 1.

Independent search ƿ<â : Update the new position by applying the following equations:


(14) $$Exp=\frac{fit_{i}-\;fit_{p}}{fit_{max}-\;fit_{min}}$$

(15) $$S=D\;\times \;\sqrt{\left|Exp\right|}\;\times \left(1-\frac{EF_{s}}{MaxEF_{s}}\right)$$

(16) $$\begin{array}{c} Fisher{\;}_{j,k}^{X+1}={Fisher}_{j,k}^{X}\\ \;\;\;\;+\left(Fisher{\;}_{pos,k}^{X}-Fisher_{j,k}^{T}\right)\times Exp+n_{e}\times e\times R \end{array}$$

For each of them, the range of values is (-1, 1), and Exp is the empirical analysis value that the fisherman acquired using any $pos^{th}$ ( pos  = 1 or 2 or… or M, ƿ= j) with fishermen as the point of reference. The fitness values that show the optimal and worse results for every position update, following $X^{th}$ are, respectively, $fit{\;}_{max}$ and $fit{\;}_{min}$ . The number of iterations of the fishermen's positions is represented by X .

$Fisher{\;}_{j,k}^{X}$ and $Fisher{\;}_{j,k}^{X+1}$ indicate the location of $j^{th}$ fisherman in *k*-dimension after the iterations of $X^{th}$ and ${\left(X+1\right)}^{th}$ . The random number ns is in the interval 0–1. The $j^{th}$ individual and the reference are separated by a Euclidean distance D . A random unit vector of dimension w is the  e . Identify the primary direction of travel and the distance using empirical analysis; positive guidance should be taken from the fisherman to the reference person. The range of exploration S , which is less than or equal to D , also varies according to the absolute value of Exp and the total number of evaluations (EFs) carried out at the moment.

Group capture ƿ ≥ â: Nets are used by fishermen to cooperate with one another and maximize their fishing capability. They form up in groups of three to four at random to surround suspected areas. Following is how the model and formula are defined:


(17) $$Center=mean\left(Fisher_{C}^{X}\right)$$

(18) $$\begin{array}{c} Fisher{\;}_{j,k}^{X+1}={Fisher}_{c,j,k}^{X}+n_{2}\\ \;\;\;\;\times \left({Center}_{c}-{Fisher}_{c,k}^{X}\right)+{\left(1-\frac{2\times EF_{e}}{MaxEF_{e}}\right)}^{2}\times n_{3} \end{array}$$

The c consists of three or four people whose places have not been changed. The red-orange point of the group has $Center_{c}$ as its target point. $Fisher{\;}_{j,k}^{X+1}$ and $Fisher_{c,j,k}^{X}$ indicate the state of the $j^{th}$ fisherman in group c in the k-dimension after the ${\left(X+1\right)}^{th}$ and $X^{th}$ updates. The fisherman's speed as he reaches the center $n_{2}$ is a variable that differs from individual to individual and can range between (0, 1). The move offset $n_{3}$ has a value range of (-1, 1), a value range of (-1, 1) that decreases with an increase in the number of EFs.

b) Phase of exploitation (EFs/MaxEFs ≥ 0.5): Some fish escape capture during the search phase, but fishermen employ an effective tactic that attracts stray and hidden fish to a focal point, eventually surrounding them. Fishermen are dispersed with the greatest density in the center and the lowest density at the edges. This arrangement guarantees that those in the center capture the majority of the fish population while those in the edges grab escaping fish, hence enhancing capture rates through tactical cooperation. The positions are updated using the Gaussian distribution as follows:


(19) $$\eta =\sqrt{\left(2(1-\frac{EF_{e}}{MaxEF_{e}})/{\left(1-\frac{2\;\times EF_{e}}{MaxEF_{e}}\right)}^{2}\right)}\;\;\;$$

(20) $$Fisher_{j,k}^{X+1}=Gbest+GD(0,\frac{n_{4}\;\times \eta \;\times mean\;(Fisher)-Gbest}{3})$$

- Among these, the Gaussian distribution function or GD has an overall mean of µ at 0 and an overall variance or η that grows with an increase in evaluations and approaches 0 from 1, the position of the jth fisherman after the ${\left(X+1\right)}^{th}$ update. The $mean\left(Fisher\right)\;$ is the fishermen's center matrix, which shows the average value of each dimension. A random integer $n_{4\;}$ , having a value of $\left\{1,\;2,\text{ or }3\right\}$ , is used to disperse fishermen into three ranges with Gbest representing the global optimal location.

Step 4: If the termination condition is not satisfied, repeat steps 2–4. In step 5, the final fitness and optimal positions are obtained. The local region is explored by encircling, and the independent inquiry is converted according to the catch rate into group capture, making the global exploration thorough and effective. To enable the CFO algorithm to identify the optimal solution with good robustness and efficacy, the optimal position is continually refined using group capture. The final selected features are passed to the shallow wide neural network classifier for the final AD prediction, as shown in **Figure 6**. The shallow neural network has an advantage as it requires less computational power, and it is easier to perform classification compared to machine learning and traditional multi-layered perception (MLP). This simplicity often results in faster processing times, which is crucial for timely diagnoses. The shallow neural network of this work consists of an input layer that accepts best features as input, a single hidden layer that is fully connected, and an output layer for the final classification.

## 4 Results and discussions

### 4.1 Experimental setup and performance measures

This section presents the experimental setup for the presented framework. There are two publicly available datasets, such as ADNI and MRI Alzheimer, that have been employed for the validation of the proposed framework. There is a 70:30 split between training and testing data, as mentioned in the section on the training of the proposed architecture. In the training phase, the initial learning rate was initialized through CFO algorithm that is 0.00014, momentum value of 0.7002, and batch size of 64. All of the experiments were carried out using 10-fold cross-validation. Cross-validation is performed at 10 k-folds in order to balance the computational bias and variance. Also, it increases the generalizability of performance estimates. The classifiers are chosen based on hidden layers, including neural networks such as narrow neural network (NNN), medium neural network (MNN), bi-layered neural network (BNN), SWNN, and tri-layered neural network (TNN), respectively. Each classifier performance is computed using several performance measures such as sensitivity, precision, F1-score, accuracy, and false negative rate (FNR). The simulation was run on a workstation with a 12-GB RTX 3000 graphics card and 256 GB of RAM, and all experiments were conducted using MATLAB 2023b.

### 4.2 Proposed framework results using ADNI dataset

**Figure 7:**
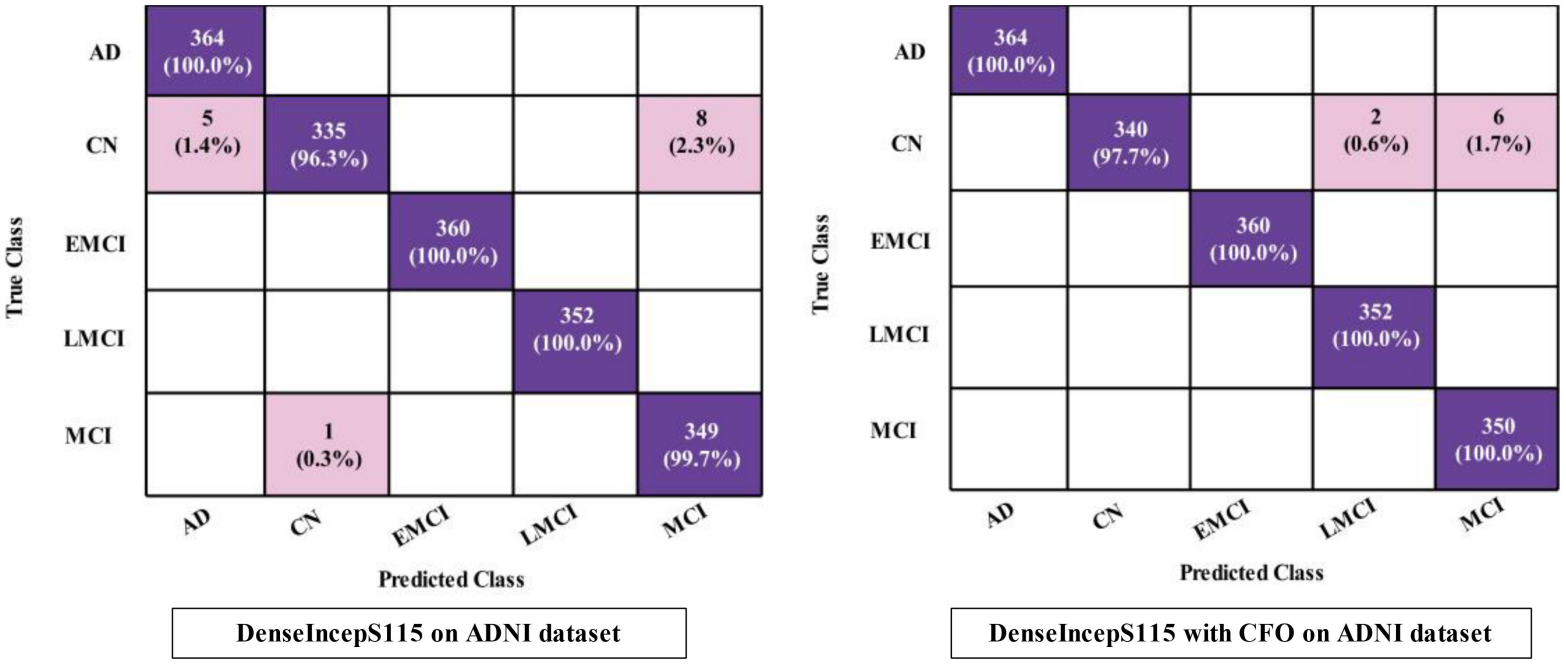
Confusion matrices of SWNN classifier for ADNI dataset.

The proposed DenseIncepS115 classification results for the Alzheimer's ADNI dataset are presented in this subsection. Features are extracted from the depth-wise concatenation layer and passed to the several classifiers including the base classifier SWNN. **Table 2** discusses the results of this experiment, whereas the maximum obtained accuracy is 99.2% for the SWNN classifier. The precision and sensitivity rate of this classifier is also 99.2%. In addition to that, the F1-score is also computed to be of the value 99.22%. The rest of the listed classifiers in this table also obtained an accuracy rate of 98.4%, 85.6%, 99.1%, and 98.5%, respectively. The obtained precision rates for these classifiers are 98.4%, 85.94%, 99.12%, and 98.52%, respectively. Hence, it is noted that the NNN, SWNN, and TNN classifiers obtained better precision rates. Time is also noted during the final classification process, as mentioned in this table. The minimum required time is 14.05 (s) for SWNN classifier, whereas the maximum consumed time is 22.03 (s) by TNN. The SWNN classifier performance can be further verified through a confusion matrix, as shown in **Figure 7**. From this figure, it can be observed that the AD class predicted 100% correctly, whereas the CN class' correct prediction is at 96.3%.

**Table 2:** Classification results of the proposed fused network DenseIncepS115 on ADNI dataset.

Classifiers	Accuracy (%)	Sensitivity (%)	Precision (%)	F1 score (%)	FNR (%)	Time (s)
NNN	98.4	98.34	98.4	98.36	1.6	14.54
MNN	85.6	85.56	85.94	85.74	14.4	17.84
BNN	99.1	99.08	99.12	99.1	0.9	20.55
**SWNN**	**99.2**	**99.2**	**99.22**	99.21	**0.8**	**14.05**
TNN	98.5	98.54	98.52	98.53	1.4	22.03

Bold denotes the highest accuracy.

The obtained accuracy and computational time are further optimized by employing a CFO algorithm that selects the best features. The results are presented in **Table 3**, showing the best accuracy of 99.5% for the SWNN classifier, whereas the time is reduced to 5.422 (s). The precision rate of this classifier is improved to 99.5% after the optimization process. In addition, the other classifiers, precision rates are 99.48%, 99.24%, 98.52%, and 97.36%, respectively. The precision rates are significantly improved for all of the classifiers after the optimization process. Moreover, time is also reduced after employing the optimization process as mentioned in this table, such as 6.623, 7.801, 8.218, and 9.503 (s), respectively. **Figure 7** (DenseIncepS115 with CFO on ADNI dataset) illustrates the confusion matrix of SWNN classifier and shows the improved prediction accuracy. Overall, the SWNN classifier performed better for this dataset using both experiments.

**Table 3:** Classification results of the proposed fused network after employing Catch Fish Optimization Algorithm on the ADNI dataset.

Classifiers	Accuracy (%)	Sensitivity (%)	Precision (%)	F1 score (%)	FNR (%)	Time (s)
NNN	99.5	99.48	99.48	99.4	0.5	6.623
MNN	99.2	99.2	99.24	99.2	0.8	7.801
**SWNN**	**99.5**	**99.54**	**99.5**	**99.5**	**0.4**	**5.422**
BNN	98.5	99.48	98.52	99.9	1.5	8.218
TNN	97.3	97.28	97.36	97.2	2.7	9.503

Bold denotes the highest accuracy.

### 4.3 Proposed results using the Alzheimer MRI dataset

**Figure 8:**
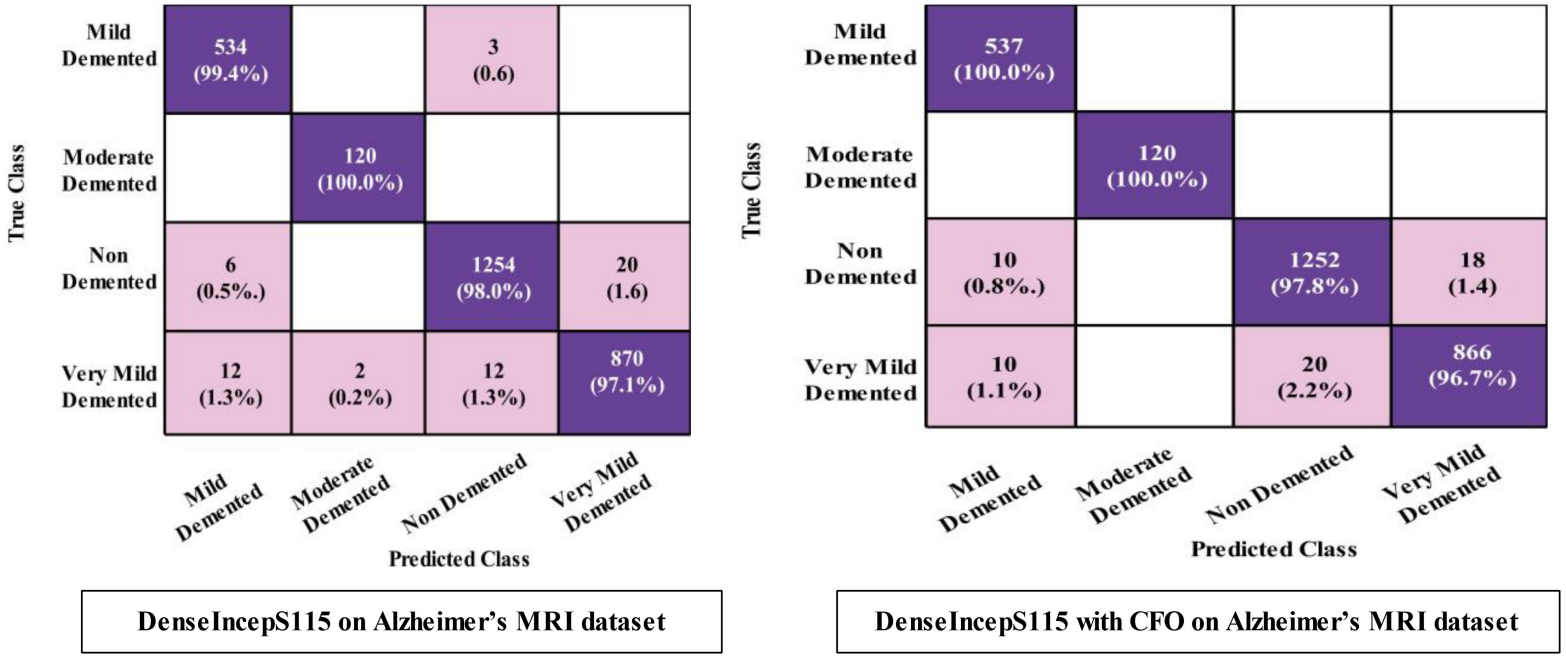
Confusion matrices of SWNN classifier for Alzheimer MRI dataset.

The prediction results for the Alzheimer MRI dataset using the proposed DenseIncepS115 architecture are presented in **Table 4**. Features are extracted from the depth concatenation layer and passed to the optimization algorithm for the selection of best features. In the first experiment, the results are computed and listed in **Table 4** for the originally deep extracted feature vector. The SWNN classifier obtained the highest accuracy of 98.1%, followed by the F1-score of 98.26%, recall rate of 98.62%, and precision rate of 97.92%, respectively. The values of these obtained measures can be confirmed by a confusion matrix, as shown in **Figure 8** (DenseIncepS115 on Alzheimer MRI dataset). The rest of the classifiers' precision rates are 96.67%, 97.27%, 96.85%, and 96.80%, respectively. Time is also noted for this experiment during the prediction process, and the minimum noted time is 10.2 (s) for the SWNN classifier, whereas the maximum consumed time is 28.7 (s) for the TNN classifier. To improve the prediction rate and reduce the computational time, we employed the CFO algorithm, and the results are presented in **Table 5**. The SWNN classifier had the highest accuracy of 98.7%, whereas the noted F1-score of 98.2% was improved compared with experiment 1 for this dataset. The recall rate is 98.6%, and the precision rate is 98.2%, respectively. These measures can be confirmed by a confusion matrix (illustrated in **Figure 8**). In this figure, it is observed that the correct prediction rate of each class has been improved after employing the optimization algorithm. Time is also noted for each classifier during the prediction step, and SWNN classifier executed the fastest with an execution time of 7.654 (s). Overall, the precision rate is improved, and the time is reduced after employing the optimization algorithm.

**Table 4:** Classification results of the proposed fused network DenseIncepS115 on the Alzheimer MRI dataset.

Classifiers	Accuracy (%)	Sensitivity (%)	Precision (%)	F1 score (%)	FNR (%)	Time (s)
NNN	96.9	97.72	96.67	97.19	3.1	23.71
**SWNN**	**98.1**	**98.62**	**97.92**	**98.26**	**1.9**	**10.2**
MNN	97.1	97.95	97.27	97.60	2.9	11.8
BNN	96.8	97.82	96.85	97.33	3.2	18.9
TNN	96.7	97.65	96.8	97.22	3.3	28.7

Bold denotes the highest accuracy.

**Table 5:** Classification results of the proposed fused network after employing Catch Fish Optimization Algorithm on the Alzheimer MRI dataset.

Classifiers	Accuracy (%)	Sensitivity (%)	Precision (%)	F1 score (%)	FNR (%)	Time (s)
NNN	97.6	98.3	97.95	97.9	1.7	9.121
MNN	97.4	98.2	97.8	97.2	1.8	8.622
**SWNN**	**98.7**	**98.6**	**98.2**	**98.2**	**1.4**	**7.654**
BNN	97.6	98.3	97.4	97.9	1.7	9.732
TNN	96.7	97.7	96.6	97.1	2.3	12.977

Bold denotes the highest accuracy.

### 4.4 Discussion

A detailed discussion of the proposed architecture is described in this section in terms of ablation studies and comparison with state-of-the-art (SOTA) techniques. The proposed AD prediction framework that consists of two phases—training phase and testing phase—is illustrated in **Figure 2**. The proposed architecture is trained on AD MRI image datasets such as ADNI and Alzheimer MRI. The hyperparameters of the proposed architecture are initialized using the BO algorithm instead of manual selection. In the testing phase, features are extracted in the first phase and passed to the classifiers, and the results are noted in **Tables 2**, **4**. In order to improve the precision rate and reduce the computational time, the CFO algorithm has been employed, and the results are given in **Tables 3**, **5**. The SWNN classifier shows the improved performance that can be confirmed through confusion matrices, as illustrated in **Figures 7**, **8**. To further validate the proposed architecture, we performed several ablation studies as follows:

Ablation study 1: In the first ablation study, we performed four experiments for each dataset. In the first experiment, features are extracted from the self-attention layer of the Proposed InceptionSA Module and passed to classifiers. In the second experiment, the Proposed DenseSA Module is opted, and self-attention layer features that are fused at the network level in the third experiment are extracted. In the last experiment, optimization is performed on the fused network. **Tables 6**, **7** present the precision values of these experiments for the selected datasets—ADNI and Alzheimer MRI. In **Table 6**, the ADNI dataset precision values are presented for each experiment. For the Proposed InceptionSA Module, the highest precision value is 95.20% which was achieved by the SWNN classifier. The precision rate is improved by the Proposed DenseSA Module to 96.16%, which is further improved in the network-level fusion step (99.21%). In the optimization, the precision rate is a little decreased, but, overall, the SWNN classifier performed well and the fusion process shows strength.

**Table 6:** Precision-based analysis of the proposed architecture performance using the ADNI dataset.

Classifier	Deep learning model				Performance measure
	Proposed InceptionSA module	Proposed DenseSA module	Network fusion	Optimization	Precision Rate (%)
NNN classifier	✔				92.46
		✔			93.50
			✔		98.40
				✔	99.48
MNN classifier	✔				90.20
		✔			91.04
			✔		85.74
				✔	99.24
BNN classifier	✔				93.62
		✔			94.24
			✔		99.10
				✔	**99.50**
**Shallow WNN classifier**	✔				**95.20**
		✔			**96.16**
			✔		**99.21**
				✔	98.52
TNN classifier	✔				93.06
		✔			94.26
			✔		98.53
				✔	97.36

Bold denotes the highest accuracy.

**Table 7:** Precision-based analysis of the proposed architecture performance using the Alzheimer MRI dataset.

Classifier	Deep learning model				Performance measure
	Proposed InceptionSA module	Proposed DenseSA module	Network fusion	Optimization	Precision rate (%)
NNN classifier	✔				91.60
		✔			92.80
			✔		96.67
				✔	97.95
MNN classifier	✔				91.04
		✔			92.30
			✔		97.27
				✔	97.80
BNN classifier	✔				92.50
		✔			93.50
			✔		96.85
				✔	97.40
Shallow WNN classifier	✔				**93.60**
		✔			**94.76**
			✔		**97.92**
				✔	**98.20**
TNN classifier	✔				92.10
		✔			93.16
			✔		96.80
				✔	96.60

Bold denotes the highest accuracy.

**Table 7** presents the obtained precision rates for each experiment using the Alzheimer MRI dataset. In this table, the Proposed InceptionSA Module obtained the highest precision rate of 93.60%, whereas the Proposed DenseSA Module improved the precision value to 94.76%. The precision rate of this experiment is further improved in the fusion step to 97.92%, which is a significant strength of this experiment. After the optimization process (experiment 4), the highest obtained precision rate is 98.20% by the SWNN classifier. Hence, the SWNN classifier shows the improved precision rate for the proposed fused network architecture and optimization algorithm.

**Figure 9:**
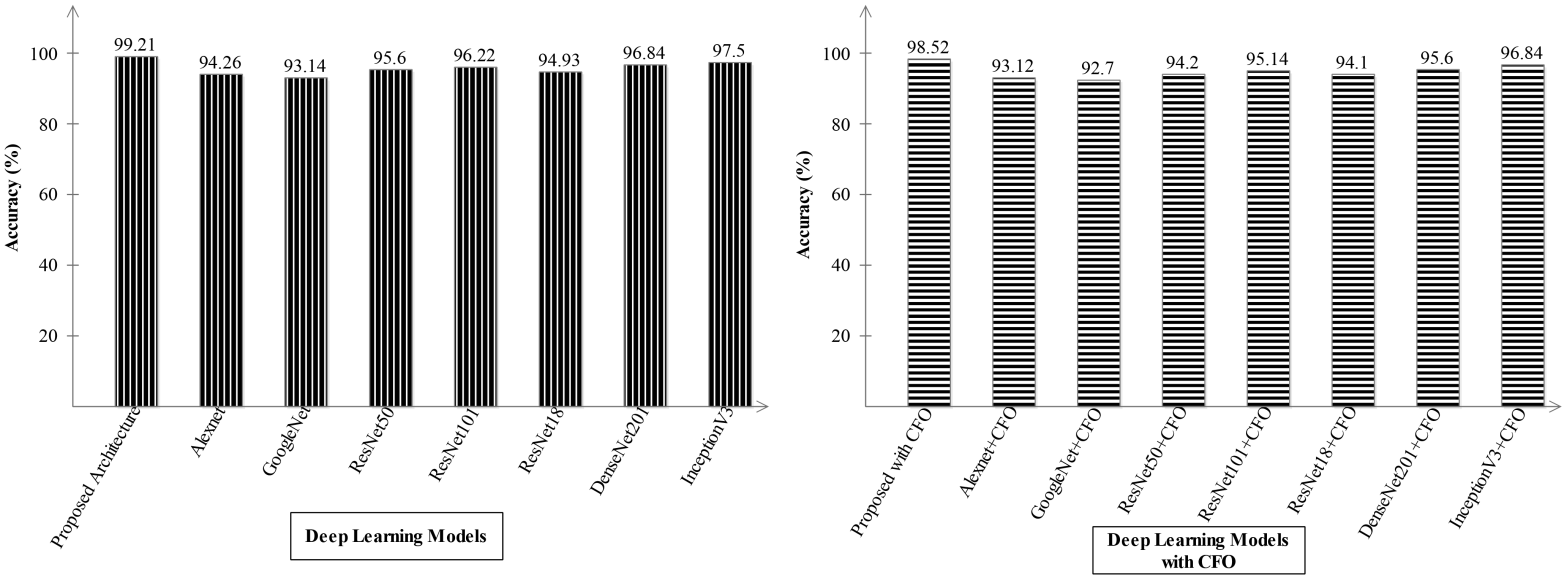
Analysis in the base of accuracy for the proposed architecture and state-of-the-art pre-trained models using ADNI dataset. The results are computed with and without optimization algorithm.

**Figure 10:**
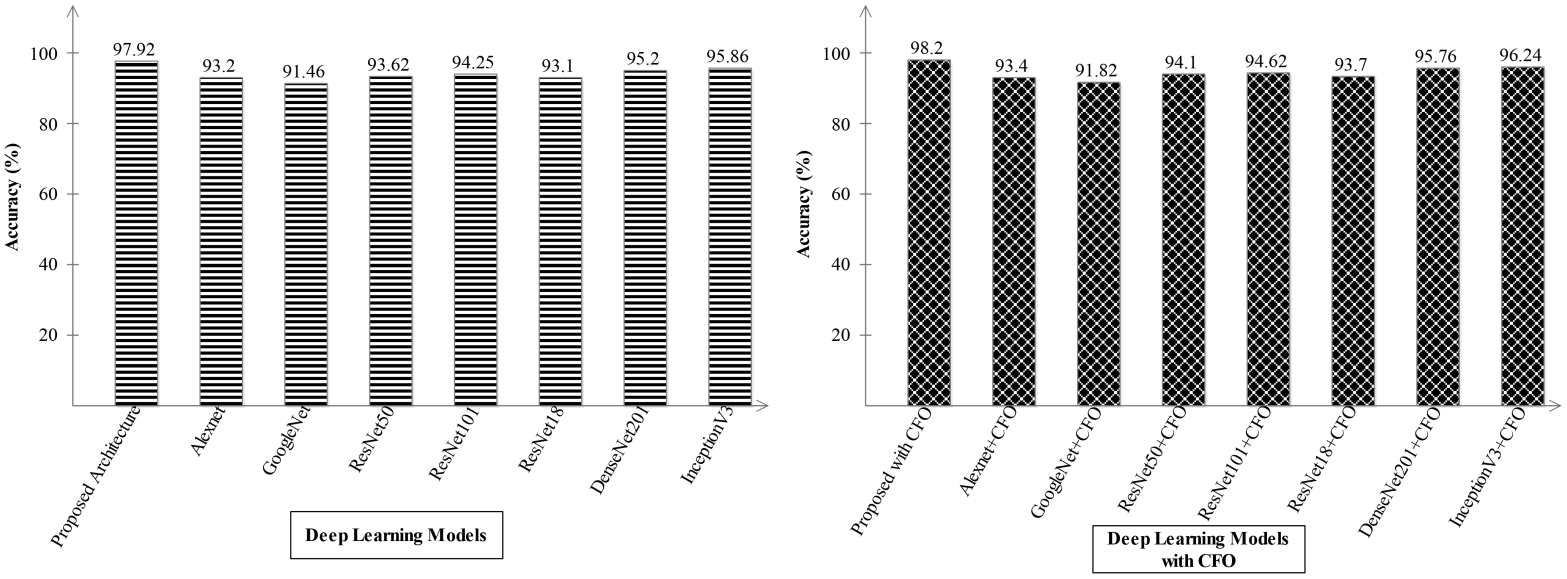
Analysis in the base of accuracy for the proposed architecture and state-of-the-art pre-trained models using the Alzheimer MRI dataset.

Ablation study 2: In the second ablation study, we compared the proposed fused network with several pre-trained deep learning architecture including Alexnet, GoogleNet, Resnet, and InceptionV3. **Figures 9**, **10** illustrate the detailed comparison of several pre-trained models with the proposed architecture based on the precision value. In **Figure 9**, the comparison is conducted in two different experiments for ADNI dataset: (i) precision value obtained on the original deep architectures and (ii) precision value is computed after employing the optimization algorithm on features extracted from these deep models. The proposed architecture obtained an improved precision rate of 99.21% and 98.52%, respectively, for both experiments. **Figure 10** shows the precision-based analysis for Alzheimer MRI dataset. In this figure, the proposed architecture obtained an improved accuracy of 97.92% and 98.2%, respectively, for both experiments performed.

### 4.5 Comparison with SOTA

**Figure 11:**
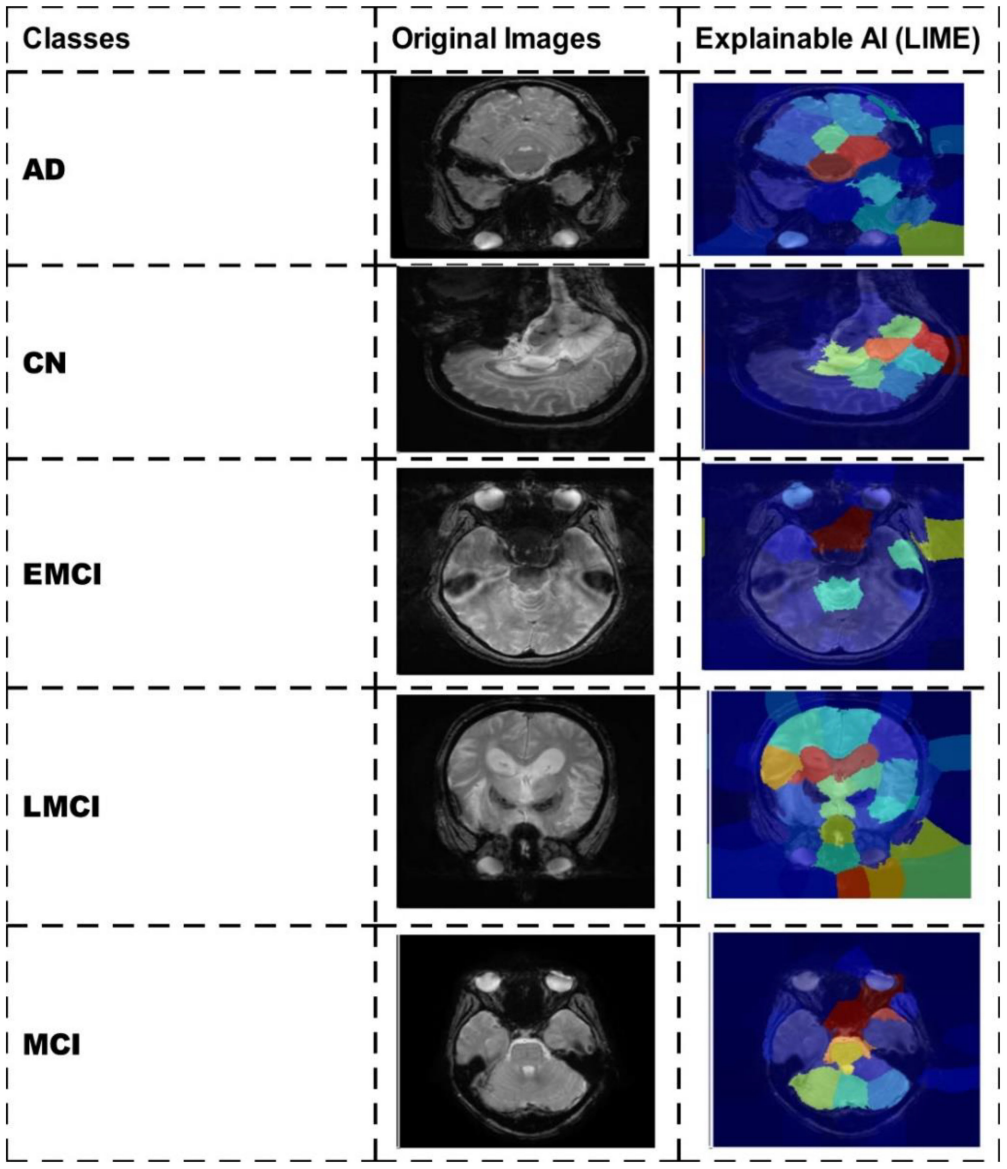
Interpretation of the proposed architecture using LIME explainable AI technique.

A detailed comparison is conducted in this section with recent state-of-the-art (SOTA) techniques. The recent SOTA techniques' accuracy value is mentioned in **Table 8**. In this table, it is observed that the obtained accuracy by Odusami et al. (42) is 73.90%, which was later improved by Raza et al. (43) and El-Assy et al. (21) to 97.84% and 99.1%, respectively. The proposed architecture obtained improved an accuracy rate of 99.5%. For the Alzheimer's Kaggle MRI dataset, Gupta et al. (44) obtained an accuracy rate of 93.7%, whereas our proposed architecture obtained an accuracy rate of 98.7%. Hence, the proposed architecture shows the improved accuracy and precision rates based on the detailed ablation studies. The proposed architecture's interpretation is also presented in **Figure 11**. In this figure, the LIME explainable AI technique is employed for the interpretation that shows the insight strength of our work.

**Table 8:** Comparison with SOTA techniques based on the selected MRI dataset of AD prediction.

S. no.	Authors/reference	Dataset	Accuracy (%)
**1.**	Odusami et al. (42)	ADNI Kaggle	73.90
**2.**	El-Assy et al. (21)	ADNI Kaggle	99.1
**3.**	Raza et al. (43)	ADNI Kaggle	97.84
**4.**	Gupta et al. (44)	Alzheimer's Kaggle MRI	93.7
**5.**	Our Proposed DenseIncepS115 CNN	Alzheimer's Kaggle MRI	98.7
**6.**	Our Proposed DenseIncepS115 CNN	ADNI Kaggle	99.5

Computational overheads: The proposed architecture is lightweight based on its number of learnable and number of hidden layers; however, the training time is almost double after the augmentation process. The proposed architecture required 253 min and 45 s; however, we also trained our model on the original images, and it takes 122 min and 12 s. The accuracy is the main difference that was noted during the training process among augmented and original images—99.1% and 99.5% (training accuracy using augmented datasets such as Alzheimer's Kaggle MRI and ADNI Kaggle) and 93.1% and 95.2% (before augmentation).

## 5 Conclusion

Alzheimer's disease (AD) is the most frequent cause of dementia, affecting millions of people globally. It causes memory loss, cognitive decline, and breakdown in day-to-day functioning, which have a major detrimental impact on people and their families. In this paper, we presented a novel deep learning architecture for AD prediction from MRI images. Dataset augmentation has been performed at the initial phase, and then a novel CNN architecture called DenseIncepS115 that is based on the fusion of two modules—Proposed InceptionSA Module and Proposed DenseSA Module—was designed. The hyperparameters of the proposed architecture are initialized using BO instead of manual selection. The trained model is later validated in the testing phase, whereas the depth concatenation layer features are extracted and optimized using the CFO algorithm. The selected features are passed to a shallow wide neural network classifier and obtained improved accuracy levels of 98.7% and 99.5%, respectively, for the selected datasets. Based on the detailed results, analysis, and ablation studies, we conclude with the following points:

- Using data argumentation step, we overcome the problems of small data samples and class imbalance.
- The proposed InceptionSA extracted information from the multiscales that was further improved in the self-attention module.
- The proposed DenseSA module improved the weights information of an image that, in return, improved the prediction accuracy and reduced the computational time.
- The proposed network-level fused architecture improved the prediction accuracy that was further optimized using the CFO algorithm. The CFO algorithm improved the precision rate and reduced the computational time.
- Model interpretation through LIME and GRAD-CAM shows how accurately the proposed architecture is trained on selected datasets for AD prediction.

Hence, the well-being of Alzheimer's disease patients may benefit from improved AD diagnosis, which is one way in which this study benefits the scientific community. In terms of accuracy and prediction, the proposed framework outperforms the current approaches. In the future, the current work shall be shifted to vision transformers.

## Data Availability

The original contributions presented in the study are included in the article/supplementary material. Further inquiries can be directed to the corresponding authors.
